# Mouth gape determines the response of marine top predators to long-term fishery-induced changes in food web structure

**DOI:** 10.1038/s41598-018-34100-8

**Published:** 2018-10-25

**Authors:** Massimiliano Drago, Valentina Franco-Trecu, Angel M. Segura, Meica Valdivia, Enrique M. González, Alex Aguilar, Luis Cardona

**Affiliations:** 1 IRBio and Department of Evolutive Biology, Ecology and Environmental Sciences, University of Barcelona, Av. Diagonal 643, 08028 Barcelona, Spain; 2 Department of Ecology and Evolution, Faculty of Sciences, Universidad de la República, Iguá 4225, 11400 Montevideo, Uruguay; 3 Modelization and Analysis of Natural Resources (MAREN), Centro Universitario Regional Este (CURE), Universidad de la República, Ruta 9, km 208, 27000 Rocha, Uruguay; 4 National Museum of Natural History (MNHN), 25 de Mayo 582, 11000 Montevideo, Uruguay

## Abstract

Here, we analyse changes throughout time in the isotopic niche of the Franciscana dolphin (*Pontoporia blainvillei*), the South American fur seal (*Arctocephalus australis*) and the South American sea lion (*Otaria flavescens*) from the Río de la Plata estuary and adjacent Atlantic Ocean to test the hypothesis that fishing may modify the diet of small-gape predators by reducing the average size of prey. The overall evidence, from stable isotope and stomach contents analyses, reveals major changes in resource partitioning between the three predators considered, mainly because of an increased access of Franciscana dolphins to juvenile demersal fishes. These results are consistent with the changes in the length distribution of demersal fish species resulting from fishing and suggest that Franciscana dolphin has been the most benefited species of the three marine mammal species considered because of its intermediate mouth gape. In conclusion, the impact of fishing on marine mammals goes beyond the simple reduction in prey biomass and is highly dependent on the mouth gape of the species involved.

## Introduction

Human activities have impacted most coastal ecosystems around the globe, with fishing as a main actor in this process^1,2^. This is because fishing usually pre-dated any other anthropogenic impacts and often overpassed them in relevance^1–5^. Although fishing may alter every ecosystem component, its impact on megafauna has been disproportionate^6^ not only because large species and large individuals are preferred targets^7,8^, but also because megafauna may be liable of experiencing high levels of bycatch^9^ and reduced food availability due to competition with fisheries^10^.

It should be noted, however, that fishing modifies not only the abundance of the potential prey of megafauna, but also the size spectrum of the community^7,8^. Aquatic predators are usually gape-limited, although exceptions exist, and cannot exploit the whole prey population when this is dominated by the older and larger age classes^11^. In this scenario, fishing may indeed increase the availability of resources for small-gape predators by reducing the average size of prey, a process further enhanced if prey are cannibal and adult predation seriously limit the population size of the younger age classes^12^.

Carnivorous marine mammals (pinnipeds and cetaceans) capture their prey through grip and tear, pierce, suction or filter feeding^13–17^. The rostral region of both pinnipeds and odontocetes is a focal region for morphological adaptation to feeding mode^14,16,17^ and largely determines the size of the prey consumed. Wide and tall skulls with a robust mandible are more suited to feed on large prey, whereas elongate skulls are more useful to capture small prey^16,17^. Certainly, species with robust skulls can also capture small prey, but the good correlation observed between skull shape and prey size^16^ suggests that species with robust skulls are more likely to be negatively affected by the selective removal of larger individuals by fisheries, whereas species with elongated skull might benefit from it.

The coastal regions of the South-western Atlantic Ocean around Río de la Plata estuary support several species of marine mammals with major differences in feeding mode and habitat. The cranial morphology of the South American fur seal (*Arctocephalus australis*) suggests pierce feeding using forward momentum^14,17^. Conversely, the Franciscana dolphin (*Pontoporia blainvillei*) has an extremely elongated skull best suited for pierce feeding using a sideways rotation of the head^17^. Finally, the skull shape of the South American sea lion (*Otaria flavescens*) is indicative of suction feeding^13,14^. They all are gape-limited^18,19^ and available information derived from the analysis of scats, stomach contents and stable isotope indicate that currently have diets primarily based on sciaenids (*Cynoscion guatucupa*, *Macrodon ancylodon* and *Micropogonias furnieri*) and anchovies (*Anchoa marinii* and *Engraulis anchoita*), although in varying proportions^20–24^. It should be noted, however, that otariid scats are biased towards the most recent meal and hence are not necessarily good proxies of their global diets. This is particularly relevant when considering differences in habitat use, as the Franciscana dolphin is a small costal marine predator foraging on-shore^25^, the sea lion is a massive, demersal predator foraging on-shore and the fur seal is a slender, pelagic predator with more off-shore feeding habits^26^.

It should be noted that, due to overfishing, the overall biomass of demersal fishes has decreased in Río de la Plata estuary and the adjacent Atlantic Ocean since the 1970s^27–29^, whereas the overall biomass of small pelagic fishes has remained rather stable^30^. The impact of demersal fishing has been particularly severe on sciaenid fishes, leading to a reduction of the total biomass^28^ and an increase in the relative abundance of the smaller size classes of species such as the stripped weakfish *C. guatucupa*^31,32^. Such a reduction might have increased food availability for small-gape predators, such as South American fur seals and Franciscana dolphins. Nevertheless, in turbid water the sweep feeding of Franciscana dolphins is much more efficient than the pierce feeding of fur seals^17^ and, hence, the former species is expected to have experienced more dramatic changes in diet than the latter.

In this paper, we analyse changes throughout time in the isotopic niche of Franciscana dolphins, South American fur seals and South American sea lions from Río de la Plata estuary and adjacent Atlantic Ocean to test the hypothesis that mouth gape determines the response of marine mammals to changes in the size spectrum of the fish community.

## Methods

### Sampling

All bone samples were obtained from the skulls of the scientific collection of the Museo Nacional de Historia Natural (MNHN) and the Facultad de Ciencias of the Universidad de la República (UdelaR) at Montevideo (Uruguay).

Mouth gape was assessed in adult fur seals (between 7 and 10 years of age) and sea lions (between 7 and 21 year of age) by measuring palate breadth after postcanines 4 and palate breadth between preorbital notches in first adult and adult dolphins (between ~3 and 6 years of age). Sample size was 15 for each sex and species group.

**Figure 1:**
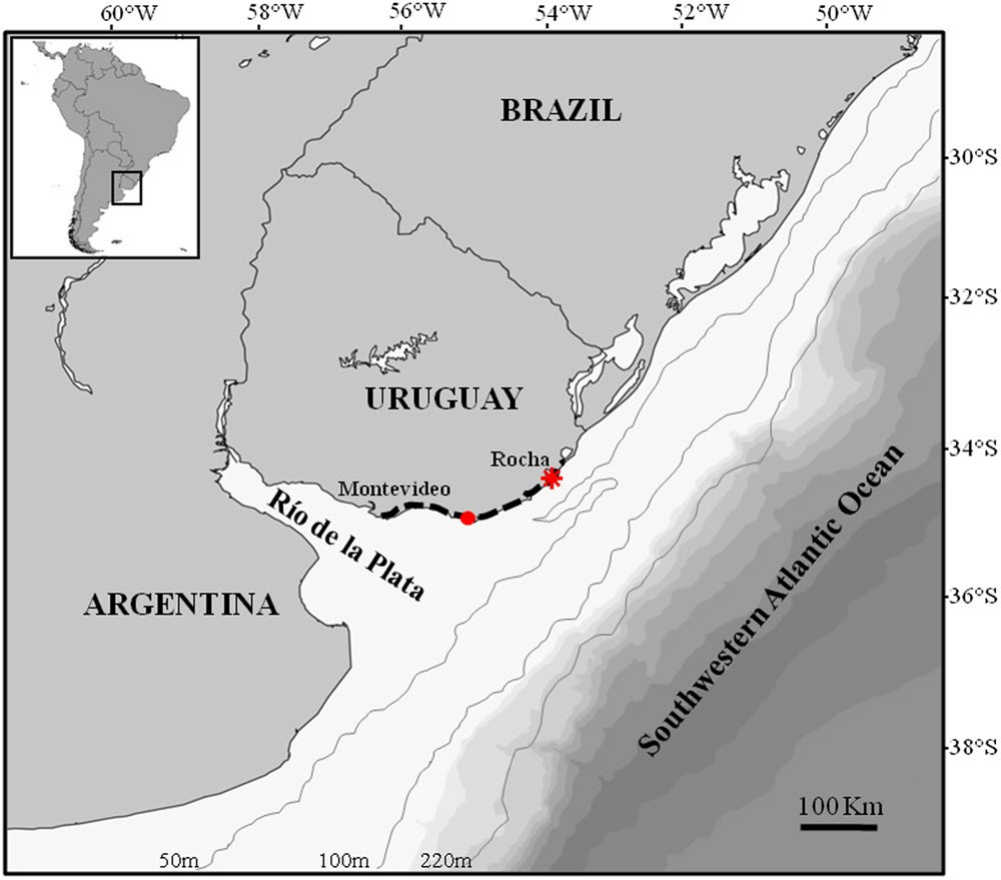
Study area and sampling locations. The dashed lines show the sampling location of Franciscana dolphin, South American sea lion and fur seal skulls from Uruguay; the red asterisk shows the location of 92% of the Franciscana dolphin skulls collected; the red dot shows the sampling location of mussel shells (*Mytilus edulis*).

Historical changes in the stable isotope ratios of sea lions and fur seals in the Río de la Plata estuary and adjacent Atlantic Ocean (Fig. 1) have already been reported by Drago *et al*.^33^ (see Supplementary Table S1). For the present study, we collected bone samples of Franciscana dolphin from males (*n* = 57), females (*n* = 45) and individuals of unknown sex (*n* = 27) which had been found stranded dead or incidentally caught by fishermen along the Uruguayan coast between 1953 and 2015. However, most of the sampled individuals (about 92%) came from the same area (Fig. 1). The individuals of unknown sex were included into the analysis to increase sample size and expand the timeline. The bone samples used for the isotopic analysis consisted of a small fragment of bone from the maxilla. In order to avoid any age-related bias, only first adult (3–5 yr old, sexually mature or in the process of maturation) and adult (6 yr or older and sexually mature) specimens were considered, as they do not differ in stable isotope ratios^34^. According to some studies^35–37^, adulthood (from ~3 to 6 yr or older) can be inferred from the standard length. The standard length of the sampled specimens ranged from 121 to 163 cm for males, from 131 to 174 cm for females and from 131 to 163 for individuals of unknown sex. Sex and standard length of specimens were initially determined during the skull collection in field.

On the basis of the year of collection, each Franciscana dolphin specimen was allocated to one of the three major periods in the recent history of marine resource exploitation in Uruguay^29,38,39^ (see Supplementary Fig. S1). The first period (1953 to 1969) was characterized by intense exploitation of fur seals, no exploitation of sea lions and negligible fishing. During the second period (1971 to 1983), both otariid species were exploited and bottom trawl fisheries were developed. During the third period (1992 to 2015), commercial hunting of fur seals and sea lions ceased and bottom trawling fisheries were fully developed (see Supplementary Fig. S1).

Samples of prey species consumed currently and in the past by Franciscana dolphins in the Río de la Plata plume and adjoining areas^21,24,40,41^ (see Supplementary Table S2) were collected from Uruguay in 2016 to determine their stable carbon and nitrogen isotope values. Samples of prey were provided by local fishermen or were collected on board by the staff of the Centro Universitario Regional Este (CURE-UdelaR). The prey species sampling was authorized by the National Council for Aquatic Resources, Ministry of Livestock, Agriculture and Fishing (DINARA, Uruguay). The stable carbon and nitrogen isotope values from some additional prey were taken from Franco-Trecu *et al*.^23,24^ (see Supplementary Table S3). Furthermore, complete fish otoliths and cephalopod beaks from 38 stomachs of Franciscana dolphins collected along the Uruguayan cost in 2009^24^, were measured with digital calipers in order to determine the length-frequency distribution of potential prey species currently consumed by Franciscana dolphins in Uruguay. Fish otoliths and cephalopod beaks were identified with the aid of published catalogues^42,43^.

All bone samples were stored dry at room temperature, whereas all samples of prey were stored in a freezer at −20 °C until analysis.

### Stable Isotope Analysis

Samples were cleaned in distilled water, dried in a stove at 60 °C for 36 h, and ground into a fine powder using a mortar and a pestle. As bone samples contain a high concentration of inorganic carbon that may add undesirable variability to δ^13^C^44^, they were treated by soaking in 0.5 N hydrochloric acid (HCl) for 24 h to decarbonise them^45^. Since HCl treatment adversely affects δ^15^N^46^, each sample was previously divided into two subsamples: one of them for carbon isotope analysis after decarbonation; and the other one for nitrogen isotope analysis without decarbonation. Furthermore, lipids were removed from all samples by a chloroform-methanol (2:1) solution^47^, as they are depleted in ^13^C compared with other molecules and may therefore lead to undesirable variability in δ^13^C values^48^. Nevertheless, given that chemical lipid extraction may affect δ^15^N values due, *inter alia*, to the unintentional removal of amino acids^49^, we extracted lipids only from the subsamples for carbon isotope analysis and used non-extracted subsamples for nitrogen determination.

Approximately 1 mg of bone and 0.3 mg of muscle from fish and crustaceans and mantle from cephalopods were weighed into tin capsules and analyzed by elemental analysis-isotope ratio mass spectrometry, specifically by means of a model FlashEA 1112 elemental analyzer (Thermo Fisher Scientific, Milan, Italy) coupled with a Delta C isotope ratio mass spectrometer (ThermoFinnigan, Bremen, Germany). All analyses were performed at the Centres Cientifics i Tecnològics of the University of Barcelona, Spain.

Stable isotope abundances are expressed in delta (δ) notation, with relative variations of stable isotope ratios expressed in per mil (‰) deviations from predefined international standards, and they were calculated as:

$${{\rm{\delta }}}^{{\rm{j}}}{\rm{X}}=[{({}^{{\rm{j}}}{\rm{X}}/{}^{{\rm{i}}}{\rm{X}})}_{{\rm{sample}}}/{({}^{{\rm{j}}}{\rm{X}}/{}^{{\rm{i}}}{\rm{X}})}_{{\rm{standard}}}]-1$$

where ^j^X is the heavier isotope (^13^C or ^15^N), and ^i^X is the lighter isotope (^12^C or ^14^N) in the analytical sample and international measurement standard^50^; international standards were the Vienna Pee Dee Belemnite (VPDB) calcium carbonate for the δ^13^C value and atmospheric nitrogen for the δ^15^N value. However, data were normalized using commercially available laboratory reference materials. For carbon, isotopic reference materials of known ^13^C/^12^C ratios, as given by the International Atomic Energy Agency (IAEA, Vienna, Austria), were used for calibration at a precision of 0.05‰. These include polyethylene (IAEA CH_7_, δ^13^C = −32.1‰), L-glutamic acid (IAEA USGS_40_, δ^13^C = −26.4‰) and sucrose (IAEA CH_6_, δ^13^C = −10.4‰). For nitrogen, isotopic reference materials of known ^15^N/^14^N ratios were used for calibration at a precision of 0.2‰. These include (NH_4_)_2_SO_4_ (IAEA N_1_, δ^15^N = +0.4‰ and IAEA N_2_, δ^15^N = +20.3‰), L-glutamic acid (IAEA USGS_40_, δ^15^N = −4.5‰) and KNO_3_ (IAEA NO_3_, δ^15^N = +4.7‰). All these isotopic reference materials were employed to recalibrate the system once every 12 samples were analyzed in order to compensate for any measurement drift over time. The raw data were normalized by the multipoint normalization method based on linear regression^51^. Furthermore, we also quantified the carbon to nitrogen (C/N) atomic ratio of each analyzed sample as a control or proxy for the data quality (e.g., adequate lipid extraction or conservation status of the isotopic signal) of the bone collagen in Franciscana dolphins and muscle and mantle in prey samples^52,53^.

### Data analyses

We compared the palate breath of the six groups (species x sex) using one way ANOVA, followed by Tukey post-hoc test.

We compared the stable isotope values (δ^13^C and δ^15^N) of Franciscana prey species between habitats (in-shore and off-shore) using a nested-ANOVA, with prey species nested within habitats.

The length of the prey identified in the stomach contents of the Franciscana dolphins was estimated by using regressions between total body length and otoliths length for fishes and between mantle length and lower rostral length for squids. For all prey species we used regressions previously published (see Table 1). Length-frequency distributions were calculated for each identified prey species, whose range of length was established according to the body size range of the samples of prey species used for the isotopic analysis.

**Table 1:** Length-frequency distribution of prey species found in stomach contents of Franciscana dolphins from Uruguay. *TL:* total body length (mm); *OL*: Otolith length (mm); *ML*: Mantle length (mm); *LRL*: lower rostral length (mm); *n*: sample size for each species; Size range: length range of the prey species found in stomach contents; bold frequency values: frequency for each length range.

Scientific name	Frequency (%)				Regressions	*n*	Size range (cm)	Source
	Length range (cm)							
	<5	5–12	12–20	>20				
**Demersal Fishes**								
*Micropogonias furnieri*	0%	10.8%	89.2%	0%	*TL* = −3.327 + 20.328 *OL*	37	9–17	^21^
*Cynoscion guatucupa*	0%	5.9%	29.4%	64.7%	*TL* = −3.217 + 19.133 *OL*	34	9–29	^21^
*Macrodon ancylodon*	39.1%	15.2%	28.3%	17.4%	*TL* = −69.177 + 28.267 *OL*	46	2–28	^21^
*Paralonchurus brasiliensis*	4.9%	2.4%	82.9%	9.8%	*TL* = −24.228 + 25.294 *OL*	41	4–23	^21^
*Urophycis brasiliensis*	0%	0%	60.9%	39.1%	*TL* = −97.681 + 36.94 *OL*	23	13–34	^21^
*Porichthys porosissimus*	0%	13.8%	65.5%	20.7%	*TL* = −8.335 + 26.734 *OL*	29	7–27	^66^
**Pelagic Fishes**								
*Engraulis anchoita*	0%	48.3%	51.7%	0%	*TL* = −7.401 + 38.88 *OL*	29	8–15	^20^
*Anchoa marinii*	8.1%	91.9%	0%	0%	*TL* = −2.199 + 28.025 *OL*	62	4–11	^20^
**Pelagic Cephalopods**								
*Loligo sanpaulensis*	0%	0%	0%	100%	*ML* = −9.31512 + 63.6316 *LRL*	16	28–45	^67^
**All prey**	**7.9%**	**28.1%**	**43.5%**	**20.5%**		**317**		

To evaluate changes in the standard length of male and female Franciscana dolphins over time, linear models were performed using the year of collection as a continuous explanatory variable and sex as a categorical covariate. We started with the most complex model, which included the interaction between explanatory variables, and subjected it to sequential, stepwise simplification by deleting the term that was furthest from being statistically significant. Comparisons between successive steps of model simplification were performed by the Akaike information criterion (AIC), selecting the model with the lowest AIC. The selected models were validated by residual analyses^54^.

The stable isotope ratios of organisms cannot be directly compared over time if temporal variations in the isotopic baseline exist^55^. The δ^15^N and δ^13^C values of the organic matrix of mussel shells (*Mytilus edulis*) collected in 1957, 1988 and 2014 at one site of the Uruguayan coast (Fig. 1) have revealed temporal changes in the isotopic baseline of the Río de la Plata ecosystem during the second half of the 20^th^ century^33^. Accordingly, the bone δ^15^N and δ^13^C values of Franciscana dolphins from different periods cannot be compared directly but have to be corrected to account for shifts in the baseline. To compute the baseline correction factor, the average stable isotope ratios of mussel shells from one period was first subtracted from those of mussel shells in the following, most recent period and the result was divided by the number of years elapsed between the two consecutive sampling years. This resulted into following time-dependent correction factors^33^: 0.0356‰ for δ^15^N and −0.0666‰ for δ^13^C per year between 1953 and 1987; and 0.0153‰ for δ^15^N and −0.0417‰ for δ^13^C per year since 1988. We used the 1957–1987 correction factor for the samples collected between 1953 and 1956, as we did not have mussel shells older than 1957. Corrected values are denoted as δ^13^C_cor_ and δ^15^N_cor_ through the text.

Once the bone isotope values were corrected to account for isotopic baseline shifts (see Table 2), we assessed the change in δ^13^C_cor_ and δ^15^N_cor_ values for male and female Franciscana dolphins over time using linear models, with year and standard length as a continuous explanatory variable and sex as a categorical covariate. The simplification, selection and validation of the model for each isotope were carried out as above-mentioned. In case that no significant difference between males and females was observed in the isotopic values (δ^13^C_cor_ and δ^15^N_cor_), further linear models would be performed pooling male and female data and incorporating individuals of unknown sex too.

**Table 2:** Stable isotope values (mean ± SD) of Franciscana dolphins during the three major periods in the recent history of marine resource exploitation in Uruguay (see Supplementary Fig. S1). *n*_1_: sample size for period; *n*_2_: sample size for sex; δ^13^C and δ^15^N: values not corrected for isotopic baseline shifts; δ^13^C_cor_ and δ^15^N_cor_: values corrected for isotopic baseline shifts. Unknown: individuals of unknown sex.

Period	*n*_1_	δ^15^N (‰)	δ^13^C (‰)	δ^15^N_cor_ (‰)	δ^13^C_cor_ (‰)	Sex	*n*_2_	δ^15^N (‰)	δ^13^C (‰)	δ^15^N_cor_ (‰)	δ^13^C_cor_ (‰)
1953–1969	16	19.2 ± 0.7	−13.3 ± 0.6	17.9 ± 0.8	−16.0 ± 0.8	Male	3	19.1 ± 1.0	−13.4 ± 0.4	17.9 ± 1.1	−16.1 ± 0.8
						Female	2	18.8 ± 1.2	−13.1 ± 0.3	17.4 ± 1.3	−16.0 ± 0.5
						Unknown	11	19.3 ± 0.5	−13.3 ± 0.7	18.0 ± 0.6	−16.0 ± 0.9
1971–1983	80	20.1 ± 0.8	−13.3 ± 0.8	19.3 ± 0.9	−15.3 ± 0.7	Male	42	20.1 ± 0.7	−13.4 ± 0.9	19.2 ± 0.7	−15.4 ± 0.8
						Female	38	20.2 ± 1.0	−13.2 ± 0.7	19.4 ± 1.0	−15.1 ± 0.6
						Unknown	0				
1992–2015	33	21.0 ± 0.7	−14.5 ± 1.1	20.8 ± 0.7	−15.0 ± 1.0	Male	12	20.8 ± 0.8	−14.1 ± 0.7	20.6 ± 0.9	−14.8 ± 0.7
						Female	5	21.2 ± 0.9	−14.1 ± 0.2	21.0 ± 1.0	−14.6 ± 0.2
						Unknown	16	21.0 ± 0.5	−14.9 ± 1.3	20.9 ± 0.5	−15.2 ± 1.2

Stable Isotope Bayesian Ellipses in R (SIBER)^56^ were used to estimate the isotopic niche width of Franciscana dolphins, South American sea lions and South American fur seals (data of the two otariid species from Drago *et al*.^33^, see Supplementary Table S1), once corrected their bone isotopic values in accordance with the isotopic baseline shifts, as well as to compare the isotopic niche space among species in the three major periods in the recent history of marine resource exploitation in Uruguay (see Supplementary Fig. S1). This allowed us to assess whether overall isotopic width of niches, overlap and trophic relationships (i.e. the relative positions of species niches in the isotopic space) among these three apex predator species of the Río de la Plata ecosystem had changed over time.

The SIBER approach is a Bayesian version of Layman metrics that can incorporate uncertainties such as sampling biases and small sample sizes into niche metrics^56^. Based on Markov-Chain Monte Carlo simulations, the SIBER approach obtains measures of uncertainty for constructing parameters of ellipses in a way similar to the bootstrap method. We used standard ellipse areas corrected for small sample size (SEA_C_) to plot the isotopic niche of each species within the isotopic space and to calculate the overlap among species. We also calculated the Bayesian standard ellipse areas (SEA_B_) to obtain an unbiased estimate of the isotopic niche width with credibility intervals. We used these two approaches because they are complementary each other^56^.

Prior to statistical analyses, normality was tested by means of the Lilliefors test, and homoscedasticity by means of the Levene test. We checked the assumptions of the statistical models by carrying out the customary residual analysis. Linear models were performed to evaluate changes in the bone isotope values as well as in the standard length of male and female Franciscana dolphins over time after having explored non-linear responses. An exploratory segmented response analysis showed that linear models better described the temporal changes in descriptive parameters than alternative models.

Data are always shown as mean ± standard deviation (SD) unless otherwise stated. All statistical analyses were carried out using the free software R^57^, and all functions for SIBER analyses were contained in the library SIAR (Stable Isotope Analysis in R)^58^.

## Results

**Figure 2:**
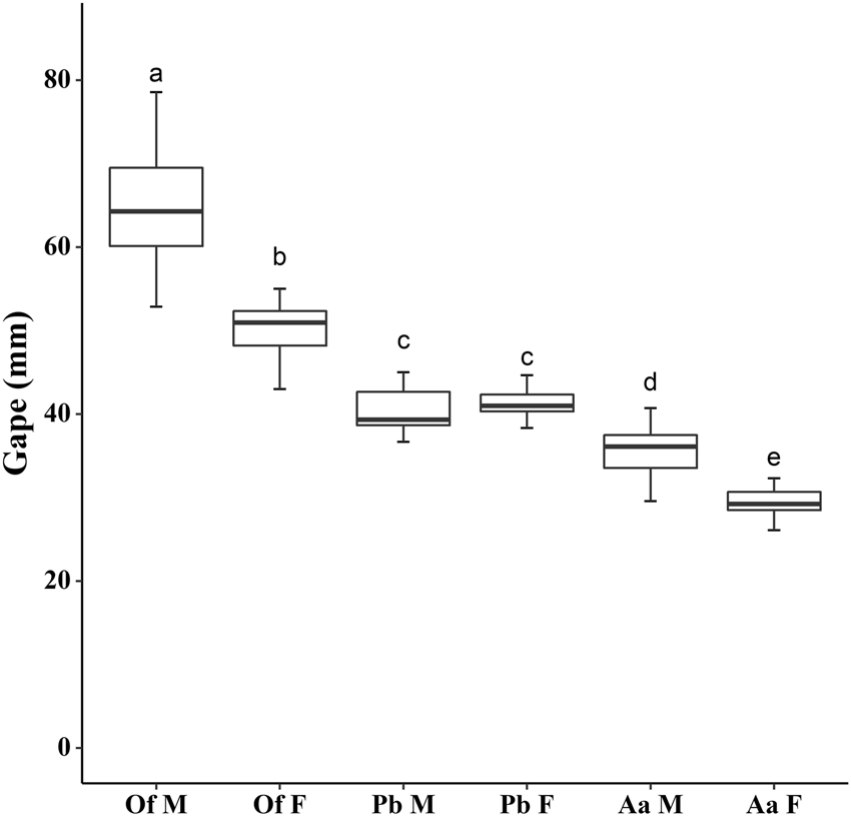
Palate breadth of males (M) and females (F) of South American sea lion (Of), Franciscana dolphin (Pb) and South American fur seal (Aa) from Río de la Plata. Species and sexes with different superscript (lower case letters) are statistically different in their mean values according to the Tukey post-hoc test. Box represents first and third quartile, line the median and whiskers 95% confidence interval of median.

Statistically significant differences existed between the palate breadth of the six groups (ANOVA; F_5,84_ = 156.928; *p* < 0.001). Male sea lions had the broadest palate breadth and female fur seals the narrowest one. Male and female dolphins did not differ in palate breadth, which was intermediate between those of female sea lions and male fur seals (Fig. 2).

The C/N atomic ratio of Franciscana dolphin bone and prey tissues ranged from 2.9 to 3.6, agreeing with the theoretical range that characterizes unaltered proteins^52,53^.

**Figure 3:**
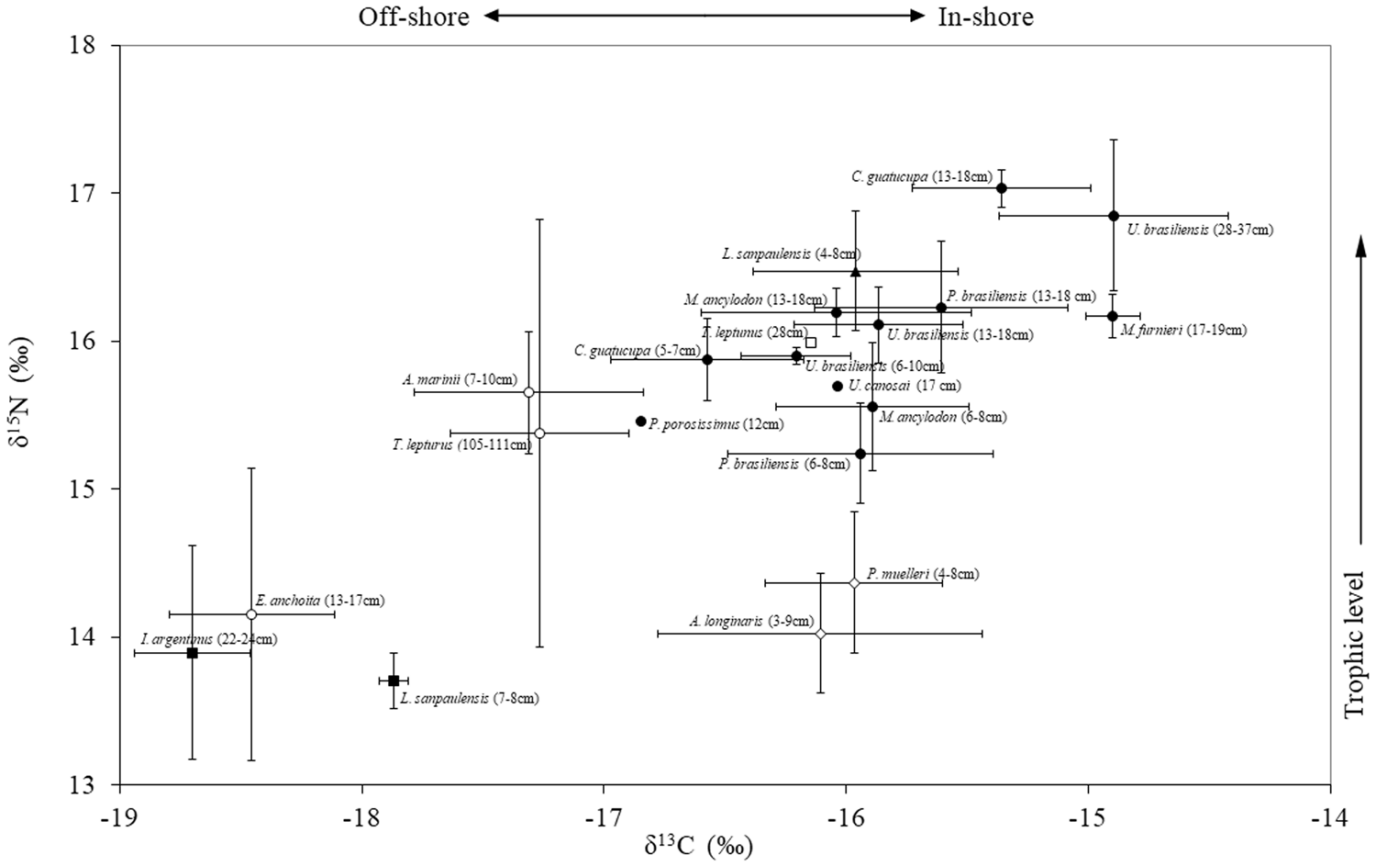
Bivariated isotopic signals (mean ± SD) of the potential prey species for Franciscana dolphins from Uruguay. In-shore pelagic fish (□), Off-shore pelagic fish (○), In-shore demersal fish (●), In-shore demersal crustaceans (◊), In-shore pelagic cephalopods (▲), Off-shore pelagic cephalopods (■). In brackets the species size range. (see original data and sample size in Supplementary Table S3).

We found that the prey of Franciscana dolphins differed statistically in their δ^13^C and δ^15^N values (nested-ANOVA; δ^13^C_model_: F_20,164_ = 19.247, *p* < 0.001; δ^15^N_model_: F_20,164_ = 41.424, *p* < 0.001), both between habitats (nested-ANOVA; δ^13^C_habitats_: F_1,164_ = 186.496, *p* < 0.001; δ^15^N_habitats_: F_1,164_ = 87.881, *p* < 0.001) and species (nested-ANOVA; δ^13^C_species_: F_19,164_ = 6.252, *p* < 0.001; δ^15^N_species_: F_19,164_ = 42.126, *p* < 0.001). This was because in-shore potential prey were usually more enriched both in δ^13^C and δ^15^N values than off-shore pelagic ones, and that the δ^13^C and δ^15^N values increased with trophic level, being medium prey (size range from 13 to 18 cm) usually more enriched both in δ^13^C and δ^15^N than small prey (size range from 5 to 10 cm) of the same species in the same habitat (see Supplementary Table S3 and Fig. 3).

A total of 317 complete prey items (301 otoliths and 16 beaks) belonging to eight fish species and one cephalopod species were found, identified and measured from the stomach contents of the Franciscana dolphins (Table 1). The length-frequency distribution of prey species currently consumed by Franciscana dolphin indicates that it mostly feeds on specimens from 12 to 20 cm although there is a certain degree of variability within each prey species, with stripped weakfish being usually larger than 20 cm (Table 1).

**Figure 4:**
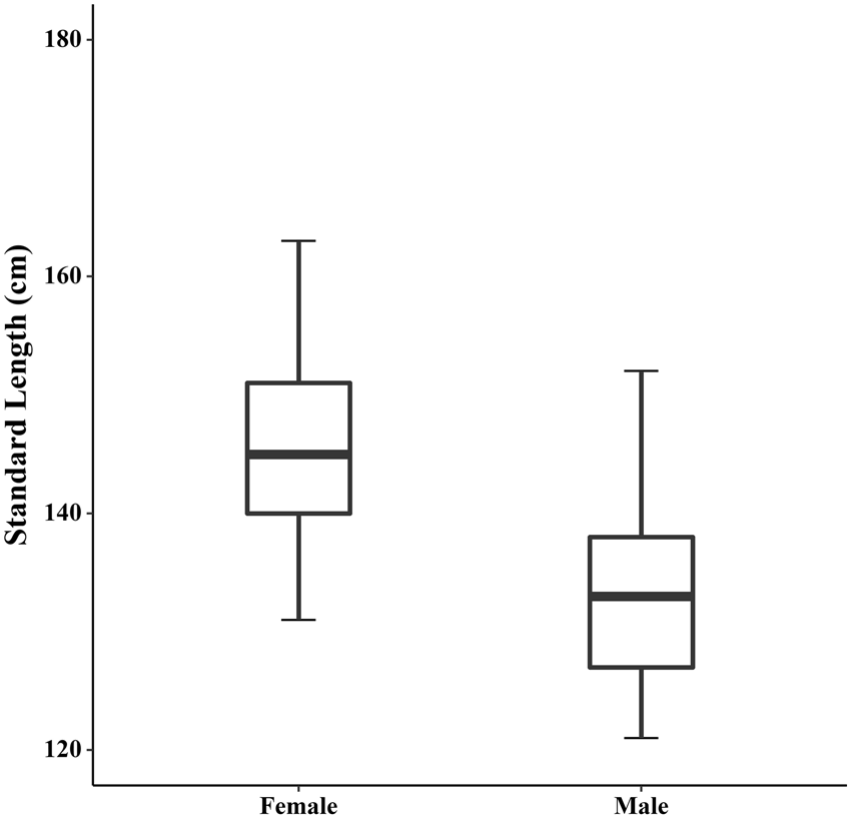
Boxplots for standard length of male and female Franciscana dolphins. Box represents first and third quartile, line the median and whiskers 95% confidence interval of median.

Linear models indicated that in Franciscana dolphins the standard length of females was significantly longer than that of males over time (Table 3 and Fig. 4). However, the linear models showed that the year of collection and the standard length were unrelated in either males or females, thus revealing no temporal trend in the standard length in both sexes (Table 3). These results confirm the reversed sexual size dimorphism observed in the Franciscana dolphin^59^ and suggest that population body size structure has remained approximately constant over time in both sexes. Furthermore, no differences existed in palate breath, as reported above.

**Table 3:** Linear models for standard length (sl) of male and female Franciscana dolphins over time. Estimates and *p-values* (in brackets) are shown for each variable. In bold we show the selected model by the Akaike Information Criterion (AIC).

Model	Intercept	Sex (male)	Year	Sex * year	AIC
sl~sex * year	221.37 (0.45)	75.76 (0.80)	−0.03 (0.79)	−0.04 (0.82)	756.89
sl~sex + year	276.14 (0.13)	−12.69 (<0.001)	−0.06 (0.47)		754.80
**sl~sex**	**146.27** (**<0.001)**	**−12.78 (<0.001)**			**752.48**

**Figure 5:**
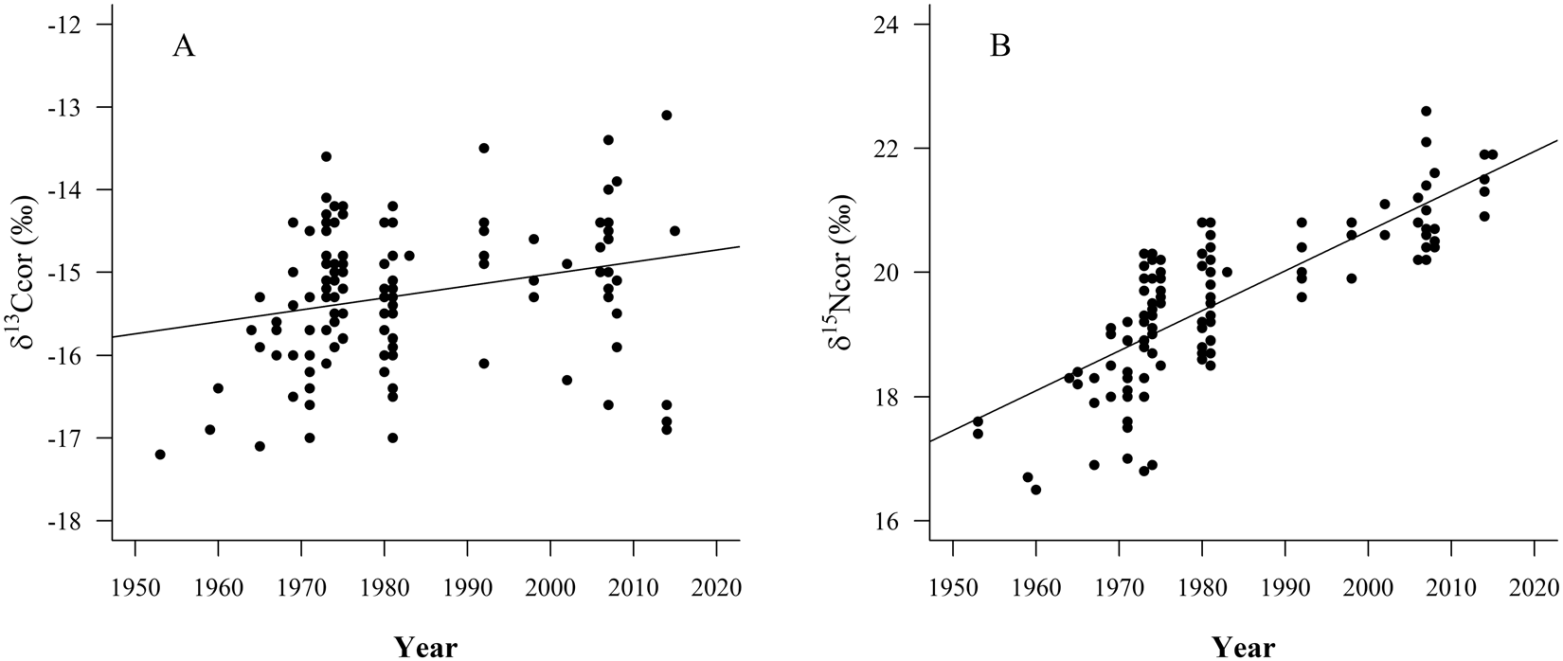
Trends of bone δ^13^C_cor_ (**A**) and δ^15^N_cor_ (**B**) values for males, females and individuals of unknown sex of Franciscana dolphins over time, once corrected in accordance with the isotopic baseline shifts, showing the fitted lines for each stable isotope (see Table 5).

Once corrected the bone isotope values for isotopic baseline shifts (Table 2), the final model adjusted for δ^15^N_cor_ showed a significant increase in Franciscana dolphin δ^15^N_cor_ values over time and a similar slope for both sexes without any effect of the standard length (Table 4). The same pattern was observed for δ^13^C_cor_ (Table 4). Furthermore, the models indicated that the average δ^13^C_cor_ and δ^15^N_cor_ values of male and female Franciscana dolphins did not differ significantly (Table 4). Accordingly, we fitted a new linear model both for δ^15^N_cor_ and for δ^13^C_cor_ pooling the isotopic data of males, females and individuals of unknown sex. This latter model showed a significant increase over time both for δ^15^N_cor_ and δ^13^C_cor_ without any effect of the standard length, although the slope of the time-δ^13^C_cor_ function was smaller than that of δ^15^N_cor_ (Table 5 and Fig. 5). We conducted 500 stratified bootstrap samples for each isotope (δ^13^C_cor_ and δ^15^N_cor_) vs year to assess the effect of variable sample size across time. For each of the three defined time periods, we sampled with replacement 15 points and estimated the slope of the relationship between stable isotopes and year. The distribution of bootstrap-estimated slopes included the value estimated in the original model with all data, suggesting that the unequal temporal distribution of samples in the data set was not biasing the model.

**Table 4:** Linear models for bone δ^13^C_cor_ and δ^15^N_cor_ values of male and female Franciscana dolphins over time. Estimates and *p-values* (in brackets) are shown for each variable. In bold we show the selected model by the Akaike Information Criterion (AIC).

Stable Isotope	Model	Intercept	Year	Sex (male)	Standard length (sl)	Sex * year	AIC
δ^15^N	δ^15^N_cor_~sex * year + sl	−140.00 (<0.001)	0.08 (<0.001)	23.23 (0.46)	0.004 (0.60)	−0.01 (0.46)	250.42
	δ^15^N_cor_~sex + year + sl	−126.00 (<0.001)	0.07 (<0.001)	−0.08 (0.65)	0.004 (0.59)		249.01
	δ^15^N_cor_~sex + year	−1.24 (<0.001)	7.26 (<0.001)	−1.44 (0.36)			247.30
	**δ** ^**15**^ **N**_**cor**_ **~year**	**−1.23** (<**0.001)**	**7.22** (<**0.001)**				**245.15**
δ^13^C	δ^13^C_cor_~sex * year + sl	−61.32 (<0.001)	0.02 (0.03)	7.28 (0.79)	0.002 (0.79)	−0.01 (0.78)	227.67
	δ^13^C_cor_~sex + year + sl	−56.70 (<0.001)	0.02 (0.003)	−0.16 (0.35)	0.002 (0.78)		225.74
	δ^13^C_cor_~sex + year	−56.15 (<0.001)	0.02 (0.001)	−0.18 (0.18)			223.82
	**δ** ^**13**^ **C**_**cor**_ **~year**	**−55.12** (<**0.001)**	**0.02** (**0.001)**				**221.62**

**Table 5:** Linear models for bone δ^13^C_cor_ and δ^15^N_cor_ values of males, females and individuals of unknown sex of Franciscana dolphins over time. Estimates and *p-values* (in brackets) are shown for each variable. In bold we show the selected model by the Akaike Information Criterion (AIC).

Stable Isotope	Model	Intercept	Year	Standard length (sl)	AIC
δ^15^N	δ^15^N_cor_~year + sl	−1.08 (<0.001)	0.06 (<0.001)	0.01 (0.92)	300.20
	**δ** ^**15**^ **N**_**cor**_ **~year**	**−1.07** (**<0.001)**	**0.06** (<**0.001)**		**298.21**
δ^13^C	δ^13^C_cor_~year + sl	−44.10 (**<**0.001)	0.01 (0.006)	0.01 (0.42)	322.04
	**δ** ^**13**^ **C**_**cor**_ **~year**	**−43.92** (**<0.001)**	**0.01** (**0.001)**		**320.68**

**Figure 6:**
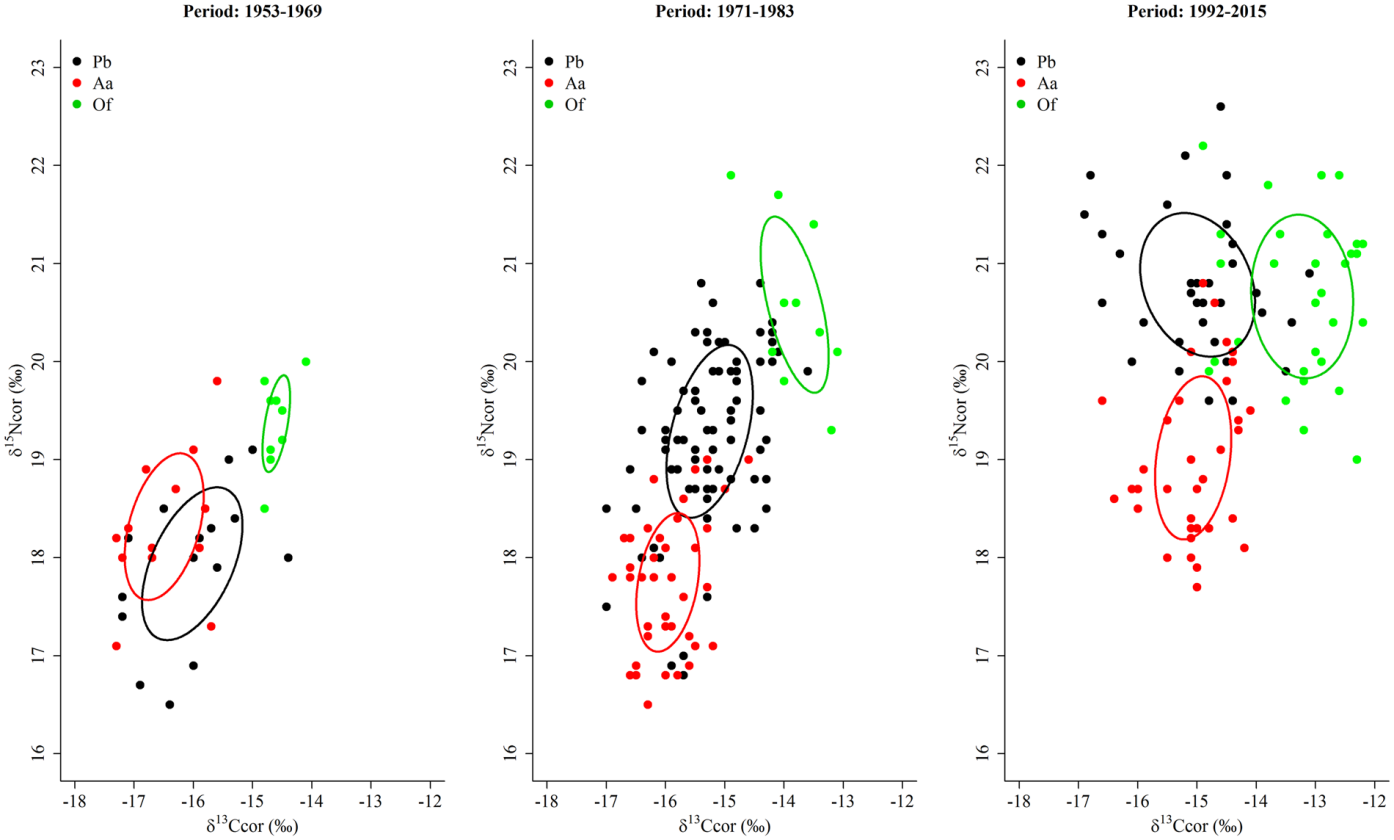
Isotopic niche areas calculated with SEA_C_ (see Table 6 for the ellipse area, credibility interval and overlap area values) for Franciscana dolphin (Pb), South American fur seals (Aa) and South American sea lions (Of) during the three major periods in the recent history of marine resource exploitation in Uruguay (see Supplementary Fig. S1). δ^13^C_cor_ and δ^15^N_cor_: values corrected for isotopic baseline shifts (see original data and sample size in Table 2 and Supplementary Table S1).

The estimated Bayesian ellipse area (calculated after correcting for isotopic baseline shifts) of Franciscana dolphins was larger than that of South American fur seals in all the considered periods (Table 6). On the other hand, it was larger than that of South American sea lions in the 1953–1969 period, but similar in the 1971–1983 period and smaller in the 1992–2015 period (Table 6). Moreover, although the Bayesian ellipses of the two otariid species did not overlap at all in any period (Fig. 6), a large overlap of the isotopic niches of Franciscana dolphins and fur seals existed in the 1953–1969 period (Table 6 and Fig. 6). Finally, although the trophic relationship (viewed as the relative positions of species niches in the isotopic space) between the two otariid species did not change over time, the distance between the ellipses of the two species was smaller in the 1953–1969 period and resembled that during the most recent period (Fig. 6). However, the trophic relationship between Franciscana dolphin and the two otariid species changed over time; Franciscana dolphins increased their trophic level throughout time and currently forage at the same trophic level than the sea lions although the Bayesian ellipses of the two species do not overlap because they differ in δ^13^C values (Fig. 6).

**Table 6:** Bayesian standard ellipse areas (SEA_B_) and their respective 95% credibility intervals (CI) for Franciscana dolphin (Pb), South American fur seals (Aa) and South American sea lions (Of) during the three major periods in the recent history of marine resource exploitation in Uruguay (see Supplementary Fig. S1). Isotopic overlap areas between species were calculated with SEA_C_, and the respective percentage of overlap surface for each species was estimated from overlap areas (see isotopic niche areas in Fig. 6).

Period	Species	SEA_B_ (‰^2^)	95% CI (‰^2^)	Species	Overlap area (‰^2^)	% Overlap area for species	
1953–1969	Pb	2.10	1.18–3.19	Pb vs. Aa	0.56	31.12	39.03
	Aa	1.82	0.97–2.86	Aa vs. Of	0	0	0
	Of	1.13	0.50–1.91	Pb vs. Of	0	0	0
1971–1983	Pb	1.88	1.49–2.30	Pb vs. Aa	0	0	0
	Aa	1.28	0.89–1.69	Aa vs. Of	0	0	0
	Of	1.88	0.89–3.08	Pb vs. Of	0	0	0
1992–2015	Pb	2.27	1.54–3.08	Pb vs. Aa	0	0	0
	Aa	1.76	1.22–2.35	Aa vs. Of	0	0	0
	Of	2.35	1.57–3.21	Pb vs. Of	0.02	0.74	0.71

## Discussion

The overall evidence presented here shows that the Franciscana dolphin has undergone a dramatic dietary change in the Río de la Plata estuary and adjacent Atlantic Ocean waters since the 1970s; currently, the Franciscana dolphin forages at the same trophic level than the South American sea lion, despite major differences in mouth gape, feeding mode and skull morphology. Conversely, in the 1950s the trophic level of Franciscana dolphins overlapped with that of South American fur seals, a result more according to their small mouth gape. These results are consistent with previous research based on stomach contents analysis reporting increased consumption of juvenile demersal fishes by Franciscana dolphins since the 1980s, particularly stripped weakfish *Cynoscion guatucupa* and to a lesser extent king weakfish *Macrodon ancylodon* (Supplementary Table S2).

The feeding mode of Franciscana dolphins, combined with the ability to locate prey by echolocation, is better suited for turbid water than that of fur seals^17,60^, which probably explain why Franciscana dolphins have undergone a more dramatic shift in isotopic niche than the latter. However, it is worth mentioning that the dramatic shift observed in the isotopic niche of Franciscana dolphin may also have been favored by the simultaneously reduction, due to by-catch, in the population size of Atlantic midshipman *Porichthys porosissimus*^61,62^, a fish species which constituted a major prey for the Franciscan dolphin until the 1980s^40,41^ (see Supplementary Table S2).

The absence of sex-related differences in palate breadth is another remarkable difference between Franciscana dolphins and the two otariid species. As a result, adults of both sexes fill the same trophic niche in the Franciscana dolphin^21,34^,^this study^ but fill different niches in the other two species^33^. In any case, the ellipses of the three species have a similar surface and hence the degree of individual specialization is comparable in the three species, despite the absence of sex-related differences in Franciscana dolphins.

It should be stressed that temporal changes in the topology of consumers in the isospace are independent of changes in the isotope baseline and hence particularly robust. On the contrary, changes throughout time in the δ^13^C and δ^15^N values of individual species are highly sensitive to the accuracy of the reconstructions of temporal changes in the isotope baseline^55^. The δ^13^C and δ^15^N values at the base of the food web may change throughout time for a number of reasons and the stable isotope ratios of the organic matter trapped in the mineral matrix of mollusk shells offer one of the few alternatives to identify and correct for shifts in the isotope baseline in the absence of tissue samples from ancient prey^55,63^.

Here, we have used the correction factors for δ^13^C and δ^15^N derived from a previous study using mussel shells to reconstruct temporal shifts in the isotope baseline of Río de la Plata ecosystem from 1957 to 2014^33^. Certainly, mussel sampling was limited to a single locality and discrete time bins, which might have hindered its representatively both at the spatial and temporal scales. Nevertheless, the δ^15^N_cor_ values of Franciscana dolphins consistently increased through time after accounting for the baseline shifts, in accordance with the topological analysis above reported and also with the results from stomach contents analysis (see Supplementary Table S2). Thus, the overall evidence is consistent with a major change in the trophic ecology of this species since the 1970s, despite the caveats about reconstructing shifts in the isotope baseline.

Sciaenids and anchovies are major dietary items for the three marine mammal species considered here, although in varying proportions^20–24^. These three top predator are gape-limited^18,19^ and certainly cannot prey on very large fishes, as shown by available data. The average size of the anchovies consumed by all them is broadly similar and always smaller than 15 cm^22^,^this study^, which is hardly surprising considering the adult size (average max length: 15.5 cm) of those species^64^. Conversely, the average size of stripped weakfish eaten by sea lions is 24.2 cm, of those consumed by fur seals is 20.5 cm and of those eaten by Franciscana dophins is 20.9 cm, although stripped weakfish can reach 65 cm^22^^,this study^. Certainly, these data have been collected after fishing resulted into major changes in the size structure of the stripped weakfish population and hence a low abundance of large specimens^32^, but they are indicative of the limitations imposed by mouth gape on prey selection.

The stripped weakfish is particularly relevant to explain the dietary shift observed in the Franciscana dolphin, because stomach contents analysis had previously revealed an increasing contribution of this species to the diet of the Franciscana dolphin since the onset of the fishing industry^40,41^ (see Supplementary Table S2). We lack such a temporal resolution for the South American fur seal and sea lion, but currently stripped weakfish is an important prey for both otariid species^20,22–24^. Interestingly, the landings of stripped weakfish were dominated by individuals larger than 30 cm at the onset of commercial exploitation, but average individual size decreased thereafter^32^. These results reveal a prevalence of the larger size classes prior to the development of demersal fishing in the 1970s, which probably limited dramatically the access of fur seals and Franciscana dolphins to most of the stripped weakfish biomass. This point is further supported by the prevalence of the Atlantic midshipman *Porichthys porosissimus*, a benthic species less than 30 cm long, in the diet of the Franciscana dolphin before the onset of fishing (see Supplementary Table S2), thus confirming its inability to exploit larger fishes.

However, the development of bottom trawling caused a major reduction in the average length of stripped weakfish in the region^31,32^ and currently the population in Río de la Plata estuary and the adjacent Atlantic Ocean is dominated by individuals less than 20 cm^31^. It is worth to note that the high prevalence of cannibalism in stripped weakfish^65^ has probably exacerbated the increase in juvenile abundance as a result of adult exploitation. This might have resulted into a higher availability of stripped weakfish for small-gape predators despite a reduction in the total biomass of stripped weakfish^28^, as far as the biomass of the smaller size classes might have increased. In this regard, the first major increase in the δ^15^N values of the Franciscana dolphin was observed during the early 1970s, immediately after the onset of the bottom trawling fishery and might reveal a fast change in the size structure of the stripped weakfish population. Certainly, stable isotope analysis alone lacks resolution to identify this kind of fine-scale changes, but in this case stomach contents analysis and stable isotope analysis represent two independent lines of evidence pointing in the same direction.

Independently on the exact details of the dietary shift reported here, the main conclusion is the impact of fishing on marine mammals goes beyond the simple reduction in prey biomass, is species specific and highly dependent on the mouth gape and foraging ecology of the considered species.

## Electronic supplementary material

Supplementary Information

## Data Availability

Data available from the University of Barcelona Digital Repository http://hdl.handle.net/2445/125380.
