# Supplementary material for: Mouth gape determines the response of marine top predators to long-term fishery-induced changes in food web structure
Source: Sci Rep. 2018 Oct 25;8:15759. doi: 10.1038/s41598-018-34100-8 (PMC6202337; doi:10.1038/s41598-018-34100-8)
Supplement: Supplementary file 1 — Supplementary Information [file 41598_2018_34100_MOESM1_ESM.pdf]

Mouth gape determines the response of marine top predators to long-term fishery-induced changes in food web structure

Massimiliano Drago, Valentina Franco-Trecu, Angel M. Segura, Meica Valdivia, Enrique M. González, Alex Aguilar, Luis Cardona.

SUPPLEMENTARY INFORMATION

SUPPLEMENTARY TABLES

Supplementary Table S1. Mean and standard deviation of the stable isotope values ($\delta^{13}C$ and $\delta^{15}N$) of South American sea lions (Of) and South American fur seals (Aa) during the three major periods in the recent history of marine resource exploitation in Uruguay. n_1: sample size for species; n_2: sample size for sex; $\delta^{13}C$ and $\delta^{15}N$: not corrected for isotopic baseline shifts; $\delta^{13}C_{cor}$ and $\delta^{15}N_{cor}$: corrected for isotopic baseline shifts. (Data from Drago *et al.* [1] and available from the Dryad Digital Repository [2]).

**Period**	**Species**	n_1	$\delta^{15}N$ (‰)	$\delta^{13}C$ (‰)	$\delta^{15}N_{cor}$ (‰)	$\delta^{13}C_{cor}$ (‰)	**Sex**	n_2	$\delta^{15}N$ (‰)	$\delta^{13}C$ (‰)	$\delta^{15}N_{cor}$ (‰)	$\delta^{13}C_{cor}$ (‰)
1953-1969	Of	9	21.0 ± 0.3	-11.2 ± 0.3	19.4 ± 0.5	-14.6 ± 0.2	Male	4	21.1 ± 0.2	-11.2 ± 0.2	19.6 ± 0.3	-14.5 ± 0.2
							Female	5	20.9 ± 0.4	-11.1 ± 0.4	19.2 ± 0.5	-14.7 ± 0.1
	Aa	13	19.8 ± 0.7	-13.3 ± 0.6	18.3 ± 0.7	-16.5 ± 0.6	Male	10	19.9 ± 0.7	-13.4 ± 0.7	18.4 ± 0.7	-16.5 ± 0.6
							Female	3	19.5 ± 0.9	-13.1 ± 0.1	17.9 ± 0.5	-16.5 ± 0.7
1971-1983	Of	10	21.5 ± 0.8	-11.8 ± 0.6	20.6 ± 0.9	-13.8 ± 0.5	Male	3	21.6 ± 0.7	-11.4 ± 0.3	20.7 ± 0.7	-13.5 ± 0.3
							Female	7	21.4 ± 0.9	-11.9 ± 0.6	20.5 ± 1.0	-14.0 ± 0.5
	Aa	38	18.7 ± 0.7	-13.9 ± 0.5	17.7 ± 0.7	-16.0 ± 0.5	Male	19	18.8 ± 0.7	-13.9 ± 0.6	17.9 ± 0.8	-16.0 ± 0.6
							Female	19	18.5 ± 0.6	-13.8 ± 0.4	17.6 ± 0.6	-16.0 ± 0.4
1992-2015	Of	30	20.8 ± 0.8	-12.8 ± 0.9	20.7 ± 0.8	-13.2 ± 0.8	Male	15	21.1 ± 0.8	-13.4 ± 0.9	21.0 ± 0.8	-13.7 ± 0.9
							Female	15	20.5 ± 0.7	-12.3 ± 0.5	20.3 ± 0.7	-12.7 ± 0.4
	Aa	35	19.1 ± 0.8	-14.7 ± 0.6	19.0 ± 0.8	-15.1 ± 0.6	Male	20	19.5 ± 0.9	-14.4 ± 0.4	19.4 ± 0.8	-14.8 ± 0.5
							Female	15	18.7 ± 0.6	-15.1 ± 0.6	18.5 ± 0.5	-15.4 ± 0.6

Supplementary Table S2. Prey species found in stomach contents of Franciscana dolphins (*Pontoporia blainvillei*) from the Río de la Plata plume and adjoining areas during the three major periods in the recent history of marine resource exploitation in Uruguay. *n*: number of Franciscana dolphin stomachs analyzed; FO (%): frequency of occurrence; N(%): relative numerical abundance of each prey species; Other species: unidentifiable species or species with N < 1%.

Period	Sampling period	*n*	Scientific name	Common name	FO (%)	N (%)	Source
1953-1969	1969	11	**Demersal Fishes**				
			Porichthys porosissimus	Atlantic midshipman	78	46	3
			Menticirrhus sp.		43	3	3
			Urophycis sp.		29	2	3
			Macrodon ancylodon	King weakfish	29	2	3
			**Pelagic Fishes**				
			Trichiurus lepturus	Largehead hairtail	57	6	3
			Stromateus brasiliensis	Butterfish	14	2	3
			**Pelagic Cephalopods**				
			Lolliguncula brevis	Atlantic brief squid	71	36	3
			**Other species**		-	3	3
1971-1983	1980-1982	157	**Demersal Fishes**				
			Porichthys porosissimus	Atlantic midshipman	33	-	4
			Cynoscion guatucupa	Stripped weakfish	22	-	4
			Micropogonias furnieri	White croacker	5	-	4
			Macrodon ancylodon	King weakfish	5	-	4
			Umbrina canosai	Argentine croaker	2	-	4
			Urophycis brasiliensis	Brazilian codling	1	-	4
			Paralonchurus brasiliensis	Banded croaker	1	-	4
			**Pelagic Fishes**				
			Engraulis anchoita	Argentine anchovy	17	-	4
			Trichiurus lepturus	Largehead hairtail	8	-	4
			Trachurus lathami	Rough scad	3	-	4
			Stromateus brasiliensis	Butterfish	2	-	4
			**Pelagic Cephalopods**				
			Lolliguncula brevis	Atlantic brief squid	62	-	4
			**Demersal Crustaceans**				
			Artemesia longinaris	Argentine stiletto shrimp	51	-	4
1992-2015	1992-2000	110	**Demersal Fishes**				
			Cynoscion guatucupa	Stripped weakfish	84	57	5
			Urophycis brasiliensis	Brazilian codling	26	3	5
			Paralonchurus brasiliensis	Banded croaker	16	2	5
			Micropogonias furnieri	White croacker	16	2	5
			**Pelagic Fishes**				
			Trachurus lathami	Rough scad	3	2	5
			Odontesthes argentinensis	Silverside	3	2	5
			Engraulis anchoita	Argentine anchovy	39	1	5

		**Pelagic Cephalopods**				
		Loligo sanpaulensis	Brazilian squid	65	21	5
		**Demersal Crustaceans**				
		Artemesia longinaris	Argentine stiletto shrimp	32	7	5
		**Other species**		-	3	5
2009	38	**Demersal Fishes**				
		Macrodon ancylodon	King weakfish	42	23	6
		Micropogonias furnieri	White croacker	13	16	6
		Porichthys porosissimus	Atlantic midshipman	23	8	6
		Paralonchurus brasiliensis	Banded croaker	21	6	6
		Urophycis brasiliensis	Brazilian codling	13	4	6
		Cynoscion guatucupa	Stripped weakfish	26	4	6
		Umbrina canosai	Argentine croaker	2	1	6
		**Pelagic Fishes**				
		Anchoa marinii	Marinis anchovy	31	19	6
		Engraulis anchoita	Argentine anchovy	42	7	6
		Merluccius hubbsi	Argentine hake	2	1	6
		Trichiurus lepturus	Largehead hairtail	2	1	6
		**Pelagic Cephalopods**				
		Loligo sanpaulensis	Brazilian squid	10	6	6
		Illex argentinus	Argentine short-finned squid	2	1	6
		**Demersal Crustaceans**				
		Pleoticus muellieri	Red shrimp	5	3	6

Supplementary Table S3. Stable isotope values (mean ± SD) of the potential prey species for Franciscana dolphins (*Pontoporia blainvillei*) from Uruguay. *n*: sample size; Size range: length range of the prey species sampled.

Scientific name	Common name	Size range (cm)	*n*	$\delta^{13}C$ (‰)	$\delta^{15}N$ (‰)	Source
**In-shore Pelagic Fishes**						
Trichiurus lepturus	Largehead hairtail	28	1	-16.1	16.0	Present study
**Off-shore Pelagic Fishes**						
Trichiurus lepturus	Largehead hairtail	105-111	2	-17.3 ± 0.4	15.4 ± 1.4	[7]
Anchoa marinii	Marinis anchovy	7-10	8	-17.3 ± 0.5	15.7 ± 0.4	Present study
Engraulis anchoita	Argentine anchovy	13-17	6	-18.5 ± 0.3	14.2 ± 1.0	[7]
**In-shore Demersal Fishes**						
Cynoscion guatucupa	Stripped weakfish	5-7	12	-16.6 ± 0.4	15.9 ± 0.3	Present study
Cynoscion guatucupa	Stripped weakfish	13-18	5	-15.4 ± 0.4	17.0 ± 0.1	Present study
Macrodon ancylodon	King weakfish	6-8	6	-15.9 ± 0.4	15.6 ± 0.4	Present study
Macrodon ancylodon	King weakfish	13-18	4	-16.0 ± 0.6	16.2 ± 0.2	Present study
Micropogonias furnieri	White croacker	17-19	7	-14.9 ± 0.1	16.2 ± 0.1	[7]
Umbrina canosai	Argentine croaker	17	1	-16.0	15.7	[7]
Urophycis brasiliensis	Brazilian codling	6-10	3	-16.2 ± 0.2	15.9 ± 0.1	Present study
Urophycis brasiliensis	Brazilian codling	13-18	13	-15.9 ± 0.3	16.1 ± 0.3	Present study
Urophycis brasiliensis	Brazilian codling	28-37	4	-14.9 ± 0.5	16.9 ± 0.5	[7]
Paralonchurus brasiliensis	Banded croaker	6-8	8	-15.9 ± 0.5	15.2 ± 0.3	Present study
Paralonchurus brasiliensis	Banded croaker	13-18	9	-15.6 ± 0.5	16.2 ± 0.4	Present study
Porichthys porosissimus	Atlantic midshipman	12	1	-16.9	15.5	[6]
**In-shore Demersal Crustaceans**						
Pleoticus muellieri	Red shrimp	4-8	21	-16.0 ± 0.4	14.4 ± 0.5	Present study
Artemesia longinaris	Argentine stiletto shrimp	3-9	34	-16.1 ± 0.7	14.0 ± 0.4	Present study
**In-shore Pelagic Cephalopods**						
Loligo sanpaulensis	Brazilian squid	4-8	16	-16.0 ± 0.4	16.5 ± 0.4	Present study
**Off-shore Pelagic Cephalopods**						
Loligo sanpaulensis	Brazilian squid	7-8	2	-17.9 ± 0.1	13.7 ± 0.2	[7]
Illex argentinus	Argentine short-finned squid	22-24	2	-18.7 ± 0.2	13.9 ± 0.7	[7]

SUPPLEMENTARY FIGURES

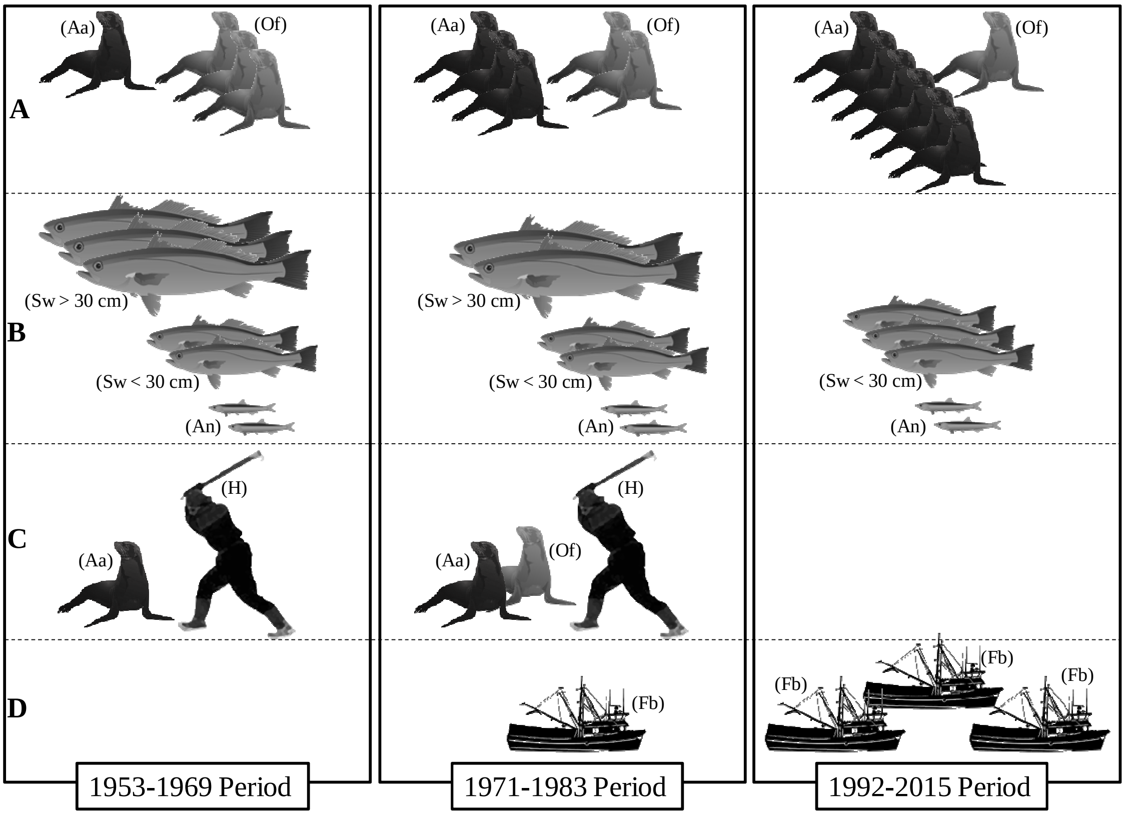

Supplementary Figure S1. Schematic representation of the three major periods in the recent history of marine resource exploitation in Uruguay [8-10]. From 1953 to 1969, there was intense exploitation of fur seals, no exploitation of sea lions and negligible fishing. From 1971 to 1983, both species of eared seals were exploited, and bottom trawlers began to operate, targeting demersal fishes. Finally, from 1992 to 2015, there was no commercial hunting of eared seals, demersal fisheries had been fully developed, and the average sizes of stripped weakfish and other demersal fish declined. (A) Relative abundance of the South American sea lion (Of) and fur seal (Aa) population; (B) Relative changes in the abundance and size distributions of the prey fish species over time (stripped weakfish (Sw) > 30 cm, stripped weakfish (Sw) < 30 cm and anchovies (An), top to bottom); (C) Commercial sealing (sea lion (Of), fur seal (Aa), hunter (H)); (D) Relative development of the industrial fishery (fishing boat (Fb)). Supplementary Figure S1 taken from the previously published article Drago *et al.* [1]; Images (not drawn to scale) from OpenClip-Art.org (https://openclipart.org) and Tracey Saxby and Dieter Tracey, Integration and Application Network, University of Maryland Center for Environmental Science (http://ian.umces.edu/imagelibrary/).

REFERENCES

1. Drago, M. *et al.* Isotopic niche partitioning between two apex predators over time. *J. Anim. Ecol.* **86**, 766-780 (2017).

2. Drago, M. *et al.* Data from: Isotopic niche partitioning between two apex predators over time. *Dryad Digital Repository* https://doi.org/10.5061/dryad.5b1cc (2017).

3. Fitch, J. E. & Brownell, R. L. Food Habits of the Franciscana *Pontoporia Blainvillei* (Cetacea: Platanistidae) from South America. *Bull. Mar. Sci.* **21**, 626-636 (1971).

4. Praderi, R. Mortalidad de franciscana, *Pontoporia blainvillei*, en pesquerías artesanales de tiburón en la costa atlántica uruguaya. *Rev. Mus. Argent. Cienc. Nat. Bernardino Rivadavia* **13**, 259-272 (1984).

5. Rodríguez, D., Rivero, L. & Bastida, R. Feeding ecology of the franciscana (*Pontoporia blainvillei*) in marine and estuarine waters of Argentina. *Lat. Am. J. Aquat. Mamm.* **1**, 77-94 (2002).

6. Franco-Trecu, V., Drago, M., Costa, P., Dimitriadis, C. & Passadore, C. Trophic relationships in apex predators in an estuary system: a multiple-method approximation. *J. Exp. Mar. Biol. Ecol.* **486**, 230-236 (2017).

7. Franco-Trecu, V. *et al.* Bias in diet determination: Incorporating traditional methods in Bayesian mixing models. *Plos One* **8**, e80019 (2013).

8. Ponce de León, A. in *Sinopsis de la biología y ecología de las poblaciones de lobos finos y leones marinos de Uruguay. Pautas para su manejo y Administración. Parte I. Biología de las especies* (eds M. Rey & F. Amestoy) 6-35 (Proyecto URU/92/003. Instituto Nacional de Pesca-Programa de las Naciones Unidas para el Desarrollo, 2000).

9. Franco-Trecu, V. *Tácticas comportamentales de forrajeo y apareamiento y dinámica poblacional de dos especies de otáridos simpátricas con tendencias poblacionales contrastantes*, Universidad de la República, Montevideo, (2015).

10. Defeo, O. *et al. Hacia un manejo ecosistémico de pesquerías: Áreas Marinas Protegidas en Uruguay*. (Facultad de Ciencias - DINARA, 2009).
